# Annotations

**Published:** 1893-07-15

**Authors:** 


### Annotations.

**Can Doctors Joke?**

The question of the capacity of doctors for joking has often been argued. Some affirm that a medical man is like a Scotchman, and requires a preliminary surgical operation On this point the following story from the first annual report of the London and Counties Medical Protection Society furnishes conclusive evidence. The story is entitled "Dr. Hilton," and is quite "Zolaistic" in its graphic realism. "In 1891," says the report, "a young man, styling himself 'E. H. Hilton, M.B., Dur.,' commenced practice at Nuneaton, in Warwickshire, and duly called upon his professional brethren. He established dispensaries in various parts of the town, and in due course set up a stylish gig, with liveried footman, &c. He promoted a Sunday class for men, gave lantern exhibitions, and otherwise sought popularity. He got married, and altogether, for eighteen months made very satisfactory progress. But Dr. Cookson had noted that 'Dr. Hilton, M.B., Dur.,' was not on the register, and communicated with the society. Particulars were ascertained, and the Registrar-General was informed that a certain death certificate had been signed by an unqualified person named Hilton. The local Registrar was instructed to demand the return of the book of certificate forms. This demand made Mr. Hilton ill with diarrhœa, &c., and as soon as he was a little recovered he disappeared, leaving no address, but several creditors." There the story ends. It is excellently told, and has an excellent moral, which moral is, that quackery, even when accompanied by Sunday-school teaching, does not pay, at any rate for very long.

**Infants and Mailcarts.**

A correspondent, writing from Margate, protests with great energy and intelligence against the use of mailcarts for very young infants. The mailcart, he says, is universal this year at Margate; and, in his judgment, it is universally bad. The complaint against these modern baby conveyances is threefold—their seats are hard, their backs are straight, and the protection against the sun is usually inadequate. "Surely," says our correspondent, "it must be bad for babies to go to sleep in an upright position." Most assuredly it is bad, and doctor after doctor has protested against the ridiculous mailcart; but all in vain. There is a fashion in these things, and mothers and nurses will follow the fashion though not only our correspondent but ten thousand hungry lions should stand growling and protesting in the way. The two most urgent objections against mailcarts for very young infants are the straight backs, which prevent lying down, and the want of adequate protection against the fierce rays of the sun. For four or five hours at a stretch, says our correspondent, nursemaids may be seen wheeling infants up and down in the blazing heat. This is not merely dangerous, it is deadly. No doubt a certain number of children will actually die of heat-stroke, whilst many more will suffer injuries to brain and nervous system, the effects of which may not be got rid of for months or years. Upon this question of heat-stroke a word may be said. Young children are very liable to heat-stroke during the heat of the mid-day hours. As a matter of fact, they ought not to be out at that time. They should be put to bed for a couple of hours at midday, and sent out for a couple of hours in the evening, say from six to eight. Of course this will be inconvenient to nurses, and therefore few mothers will have the moral courage to insist upon it. But where the mother is too feeble-minded the father should step in, and insist upon obedience to such an obviously common-sense rule. We are only at the beginning of hygienic civilisation, and servant girls of all classes constitute a barrier to progress which is almost impassable.

**Poor Pay, Bad Work.**

Australia is beating the record in the starvation of its doctors, and Australian doctors are out-Heroding Herod in their self-degradation. The Hon. Dr. Creed publicly stated the other day to the members of the New South Wales branch of the British Medical Association, that there is one suburb of Sydney in which two medical men have full charge of 10,000 patients, and are paid between them £600, or 1s. 2½d. per head per annum. This kind of thing is more or less general both in large towns and in outlying country districts. Among the persons who pay these utterly ridiculous fees are "well-to-do people, shopkeepers, manufacturers, M.P.'s, and officials," who, having joined the medical societies when they were poor, have remained in them after they have become rich because, though rich, they are not able to get rid of the native meanness of the gutter. We should like medical men to consider that this wretched pay, combined with overwhelming work has several evils of a practical kind which are of the greatest possible significance. To begin with, the system absolutely ruins the culture and the morals of the doctors who practise it, because no doctor can by any possibility do good, honest work who has so much of it to do for such poor pay. In addition to this it kills the doctors rapidly, just as omnibus and tramcar horses are quickly killed by persistent overstrain. We are certain, moreover, that the system brings medical men and the profession into profound general contempt, because the most ignorant members of the working class cannot fail to see that a man who knows better is only worthy of contempt who takes beggar's wages and does beggar's work. It is of no use blinking the facts or excusing them on the plea of the poverty of the people. The grocer does not sell his sugar for less than cost price because many of the people who buy it have small wages. The poverty of the poor may demand the consideration of the prosperous, but it can never demand the degradation of those who are bound by every consideration of education and self-respect to live clean, truthful and honest lives. A man so well educated and so decently brought up as the average doctor ought to be overwhelmed with shame when he finds himself doing medical work which is worthless and worse than worthless. Such a man is a daily fraud, a living lie. It is our hope that the self-respecting members of the profession will take up the question with a strong hand, and apply efficient remedies. We know of our personal knowledge that the things done in Australia have their counterpart in this country over very wide areas. Pity for our weak professional brethren who allow themselves to be thrust into the mire by their poverty, and an *esprit de corps* worthy of a great and cultured profession, must spur us to new and decisive efforts. That a beginning will soon be made by medical men, who feel profoundly ashamed of these things, we have every reason to hope.

